# Approach to learning and educational environment: time to rethink measurement tools in postgraduate medical training?

**DOI:** 10.5116/ijme.5c88.029d

**Published:** 2019-03-29

**Authors:** Efrain Riveros-Perez, Enow Jimenez, Thomas Cheriyan, Nicole Varela, Jorge Rodriguez, Alexander Rocuts

**Affiliations:** 1 Department of Anesthesiology and Perioperative Medicine, Medical College of Georgia at Augusta University, USA; 2 Surgery Department, Clinica de los Andes, Tunja, Colombia

**Keywords:** Learning approaches, learning environment, student perception, anesthesiology, residency training

## Abstract

**Objectives:**

To assess the correlation between perception of the learning environment and the approach to learning adopted by anesthesiology residents throughout training in an academic institution in the United States.

**Methods:**

This is a cross-sectional study involving forty-one anesthesiology residents who completed electronic forms of the Revised Two-Factor Study Process Questionnaire to assess learning approaches, and the Dundee Ready Educational Environment Measure questionnaire to assess learning environment. Convenience sampling was used with the current anesthesiology residents. Learning approaches were analyzed with a multiple regression model for correlation between total score, domains, and training level. Analysis of variance was used to assess differences in perception of the learning environment based on training level. Multivariate logistic regression was used to assess the correlation between domains of learning the environment and approaches questionnaires. Cronbach α was used to evaluate the internal consistency of responses within each domain of both questionnaires.

**Results:**

Forty-one residents completed the questionnaires. Cronbach α varied between 0.604 and 0.76 among the domains in the Study Process Questionnaire and was greater than 0.60 for the Dundee questionnaire. There was a moderate correlation between total deep approach scores and the total subjective perception of teachers scores (R^2^= - 0.507, p <0.01). There was no significant association between specific domains of Dundee and study process questionnaires and resident year of training.

**Conclusions:**

The learning approaches adopted by anesthesiology residents and the perception of the educational environment are not correlated with years of training. The DREEM and R-SPQ-2F questionnaires should not be recommended for evaluation of anesthesiology residents.

## Introduction

Anesthesiology training in the United States is oriented to the development of core competencies defined by the Accreditation Council for Graduate Medical Education (ACGME).^1^ Residency programs must enact their academic curriculum to support learning for their trainees. Although residency programs have adjusted the curriculum to the new paradigm shifts regarding teaching and competence assessment, an individual component of the equation unique to each resident determines the learning outcomes.^2^^,^^3^ The analysis of individual approach to learning addresses why and how students engage in learning and the factors that influence the selection of specific approaches.^4^

Extensive research into the phenomenon of higher learning has unearthed several theories regarding the approaches to learning based on the characteristics of each approach and the motivation of each student. Learning approaches have been divided into three distinct, but not dissimilar categories: the surface approach, the deep approach, and the strategic approach. The surface approach to learning involves the memorization and the reproduction of the desired information. The deep approach involves the organization and extrapolation of the desired information, and the strategic approach involves the academic success of the student and the highest efficiency possible.^5^

The learning environment is an important factor that influences the approach to learning adopted by a student, in aspects as diverse as content, context, and demands.^6^ Biggs proposed that the approach to learning was determined by factors inherent to each student under the influence of the educational context.^7^ This association has led researchers to postulate that modifications in the learning environment lead to better academic performance via adoption of a deep approach to learning fostered by student-centered methods. ^8^ The effect of the educational environment on learning affects undergraduate students from different disciplines and medical students.^9^^,^^10^ The approaches to learning are not static; on the contrary, the educational environment determines the transition from one approach to another;^11^ however, the relationship between learning approaches and the environment is rather complex, and is dependent on multiple factors whose interaction has not been fully elucidated.^12^  In addition, student perception rather than the learning environment itself is the variable that influences the approach to learning.^13^ Several authors have shown the association between approach and environment in relation to learning as well as its impact on academic performance in the context of general education.^14^^-^^18^ To our knowledge, the relationship between the perception of the learning environment and learning approaches have not been described in postgraduate medical trainees. We conducted this study to evaluate the effect of the perception of the learning environment on the approaches to learning adopted by anesthesiology residents, and whether the approach to learning changes as the resident progresses through training.

This study is aimed to assess the correlation of the perception of the learning environment and the approach to learning adopted by anesthesiology residents throughout training. The secondary aims are to evaluate the effect of years of training on the resident’s learning approach and to assess the association between the domains of the Dundee Ready Environment Measure (DREEM) questionnaire used to evaluate learning environment, and the learning approaches adopted by anesthesiology residents.

## Methods

### Design and participants

A cross-sectional study was conducted in forty-one residents enrolled in the anesthesiology program at Augusta University. The sample was collected by convenience to include the current anesthesiology residents enrolled in the program. The study was approved by the Institutional Board Review of Augusta University. Written consent was obtained after explaining the participants the ethical aspects of the study, including the quality and integrity of the research, respect for confidentiality and anonymity, and the independent nature of the study.  Participants were asked to complete a demographic survey and the electronic version of two questionnaires:  the Revised Two-Factor Study Process Questionnaire (R-SPQ-2F) to assess learning approaches^19^ and the Dundee Ready Educational Environment Measure (DREEM) to assess learning environment.

### Data Collection

The R-SPQ-2F instrument consists of twenty items evaluating two scales, each one composed of ten items, assessing either the superficial or the deep approach to learning. Additionally, the scales can be subdivided into two subscales of five items each, which reveal the strategies and motivations underlying the learning approaches. Responses to each item were categorized according to a 5-point Likert scale. This questionnaire evaluates the deep dimension (items 1, 2, 5, 6, 9, 10, 13, 14, 17, 18), and superficial dimension of learning approaches (items 3, 4, 7, 8, 11, 12, 15, 16, 19, 20). Biggs reported both the validity and reliability of the scale to discriminate superficial and deep learning approaches.^19^

The DREEM questionnaire consists of fifty items evaluating a range of topics in relation to the learning environment. The participant’s responses fall within five Likert categories from strongly agree to strongly disagree. The instrument evaluates five different dimensions: perceptions of learning (12 questions), the perception of course organizers (11 questions), perceptions of the atmosphere (12 questions), social self-perceptions (7 questions) and academic self-perceptions (8 questions). The total score is interpreted to allocate the participant to the following categories based on the perception of the learning environment: very poor (0-50), plenty of problems (51-100), more positive than negative (101-150), and excellent (151-200).^20^ McAleer reported on validation and reliability of the DREEM questionnaire.^21^ Demographic variables (age, gender, relationship status) and variables related to learning approach, DREEM score, and the post-graduate year from each resident were recorded.

### Procedure

The residents were invited to participate in the study in a departmental meeting. Then, an electronic link to the questionnaires as well as a consent form was sent via electronic mail. The surveys were collected ensuring anonymity. Residents received two electronic mail prompts in July 2018. Data were collected by one investigator, exported to an Excel Microsoft worksheet, and tabulated for analysis.

### Statistical analysis

Baseline characteristics including age, relationship status, and category of medical school (i.e., allopathic US medical school, osteopathic medical school or foreign medical school) were compared between junior residents at post-graduate years 1-2 (PGY-1 and PGY-2) and senior residents at PGY-3 and 4 using chi-square test. Cronbach α was used to assess internal consistency of responses within each domain of both questionnaires.^22^ A value above 0.60 was considered as satisfactory for internal consistency and values above 0.70 as good for internal consistency in a particular domain.^23^ In domains where Cronbach  α  was less than 0.60, questions were sequentially excluded beginning with the question with the lowest consistency to obtain a Cronbach α of more than 0.60.

Analysis of variance was performed to elucidate differences in the mean domain and total scores among the two residency categories (junior versus senior residents). We performed multivariate logistic regression model to assess the association between total R-SPQ-2F score, deep motive, surface motive, deep strategy, surface strategy, total surface approach, total deep approach domains, and resident category. We also performed the above analyses using the year of residency as the categorical variable i.e., PGY-1 versus PGY-2, PGY-3, and PGY-4 residents. Fisher’s least significance difference was used for post-hoc analysis. A p-value of 0.05 was considered statistically significant.

The total DREEM score and mean DREEM domain scores were standardized by dividing the total score with the number of questions included in that particular domain. Analysis of variance was performed to assess the difference in mean scores among the residency categories. Interpretation of each DREEM domain was done using modified recommendations of McAleer and colleagues to account for questions excluded to maintain internal consistency.^19^^,^^21^ Chi-square test was used to assess the association between standardized domain categories among the two residency categories (junior versus senior residents). We also performed the above analyses using the year of residency as a categorical variable, i.e., PGY-1 versus PGY-2, PGY-3, and PGY-4 residents. Fisher’s least significance difference was used for post-hoc analysis. A p-value of 0.05 was considered statistically significant.

Regarding R-SPQ-2F and DREEM correlation analyses, we assessed the correlation between each domain and total R-SPQ-2F scores with each domain and total DREEM scores using the multivariate logistic regression model with the Pearson correlation coefficient (R). An R-value of greater than 0.8 was classified as strong, 0.5-0.79 as moderate, and less than 0.5 as a weak association. A p-value of 0.01 was considered statistically significant. All statistical analyses were performed using SPSS 20 (IBM, Armonk, New York, USA).

## Results

Forty-one residents completed the DREEM and R-SPQ-2F questionnaires. The study population included 8 post-graduate year-1 (PGY-1) residents, 12 PGY-2 residents, 9 PGY-3 residents, and 12 PGY-4 residents. Age of residents varied between 27-45 years. There was no statistically significant difference in baseline characteristics except for marital status (Table 1).

### R-SPQ-2F Questionnaire

Cronbach α varied between 0.604 and 0.76 among the domains in R-SPQ-2F with question number two excluded in deep strategy domain to achieve Cronbach α of more than 0.60 (Table 2). There were no significant differences in mean scores of individual responses to questions in each domain between junior and senior residents (Table 3). There were no differences in mean domain-specific or total scores among the junior and senior residents. There was no significant association between specific domains and resident category (junior versus senior). Similarly, there was no significant association between specific domains and resident year of training (Table 4).

**Table 1:** Demographic data

Residents’ Demographics	Junior n (%)	Senior n (%)	p-value
Relationship status			
Single	10 (50)	4 (19)	0.039
Married	10 (50)	17 (81)	
Medical School			
Allopathic US medical school	6 (30)	5 (23.8)	0.804
Osteopathic Medical School	1 (5)	14 (66.7)	
Foreign medical school	13 (65)	2 (9.5)	

### DREEM Questionnaire

With respect to DREEM questionnaire, questions 1, 2 and 6 were excluded from the subjective perception of teachers domain, and question 3 was excluded from the subjective academic perception domain in order to achieve Cronbach greater than 0.60. There was no significant difference in mean scores of individual questions between junior and senior residents, except for two questions: “Last year's work has been good preparation for this year's work,” and “I seldom feel lonely.”  There was no significant association between domain category responses and resident category (junior versus senior). Similarly, there was no significant association between specific domains and resident year of training.

**Table 2:** Cronbach alpha across R-SPQ-2F and DREEM questionnaires evaluating for internal consistency within each domain

Questionnaire	Domains	Cronbach alpha
R-SPQ-2F	Deep Motive	0.746
	Deep Strategy	0.604
	Surface Motive	0.688
	Surface Strategy	0.76
DREEM	SPL	0.794
	SPT	0.646
	SPP	0.676
	SPA	0.752
	SSP	0.667

SPL, subjective perception of learning. SPT, subjective perception of teachers. SPS, subjective academic perception. SPA, subjective perception of atmosphere. SSP, social self-perception. R-SPQ-2F, Revised Study Process Questionnaire of Two Factors. DREEM, Dundee Ready Education Environment Measure.

**Table 3:** Comparison between junior and senior residents with respect to mean scores of questions within the R-SPQ-2F questionnaire

R-SPQ-2F questionnaire	Junior	Senior	p-value
Question	Mean +/- SD	Mean +/- SD	
1. I find that at times studying gives me a feeling of deep personal satisfaction.	3.2 ± 0.9	3.4 ± 1.1	0.571
2. I find that I have to do enough work on a topic so that I can form my own conclusions before I am satisfied.^*^	2.7 ± 1.2	3.3 ± 1.1	0.078
3. My aim is to pass the course while doing as little work as possible.	1.45 ± 0.7	1.7 ± 1.1	0.365
4. I only study seriously what’s given out in class or in the course outlines.	1.9 ± 0.8	1.8 ± 1.0	0.89
5. I feel that virtually any topic can be highly interesting once I get into it.	2.9 ± 1.1	3.7 ± 1.2	0.041
6. I find most new topics interesting and often spend extra time trying to obtain more information about them.	3.3 ± 0.9	3.2 ± 1.3	0.856
7. I do not find my course very interesting, so I keep my work to the minimum.	1.8 ± 0.9	1.4 ± 0.7	0.146
8. I learn some things by rote, going over and over them until I know them by heart even if I do not understand them.	2.4 ± 1.1	2.3 ± 1.0	0.845
9. I find that studying academic topics can at times be as exciting as a good novel or movie.	2.9 ± 1.1	2.4 ± 0.9	0.144
10. I test myself on important topics until I understand them completely.	3.4 ± 0.8	3.0 ± 1.0	0.132
11. I find I can get by in most assessments by memorizing key sections rather than trying to understand them.	1.9 ± 0.9	2 ± 0.8	0.709
12. I generally restrict my study to what is specifically set as I think it is unnecessary to do anything extra.	1.8 ± 1.0	1.7 ± 0.9	0.659
13. I work hard at my studies because I find the material interesting.	3.3 ± 1.1	3.5 ± 1.1	0.511
14. I spend a lot of my free time finding out more about interesting topics which have been discussed in different classes.	2.8 ± 1.0	2.9 ± 1.1	0.642
15. I find it is not helpful to study topics in depth. It confuses and wastes time, when all you need is a passing acquaintance with topics.	1.7 ± 1.0	1.7 ± 0.9	0.911
16. I believe that lecturers shouldn’t expect students to spend significant amounts of time studying material everyone knows won’t be examined.	2.3 ± 1.3	2.1 ± 0.9	0.656
17. I come to most classes with questions in mind that I want answering.	2.6 ± 0.8	2.4 ± 1.0	0.68
18. I make a point of looking at most of the suggested readings that go with the lectures.	2.8 ± 1.0	2.5 ± 1.0	0.484
19. I see no point in learning material which is not likely to be in the examination.	2 ± 1.1	2.1 ± 1.4	0.902
20. I find the best way to pass examinations is to try to remember answers to likely questions.	2.2 ± 0.9	2.1 ± 1.0	0.85

*Question 2 was removed from statistical analysis to obtain a Cronbach alpha>0.60. R-SPQ-2F, Revised Study Process Questionnaire of Two Factors.

### R-SPQ-2F and DREEM Correlation

There was a moderate correlation between total deep approach scores of the R-SPQ-2F questionnaire with the total subjective perception of teachers scores of the DREEM questionnaire (R^2^= - 0.507, p <0.01). There was no significant correlation between domains and total scores of R-SPQ-2F and DREEM questionnaire.

**Table 4:** Association of mean domain and total R-SPQ-2F scores between years of residency

Domain	PGY-1	PGY-2	PGY-3	PGY-4	p-value
Deep strategy	3.1 ± 0.5	3.0 ± 0.5	2.8 ± 1.0	3 ± 0.8	0.888
Deep motive	3.3 ± 0.6	2.8 ± 0.8	3.4 ± 0.76	2.9 ±0.7	0.178
Surface strategy	1.7 ± 0.6	2.4 ± 0.7	2.2 ± 0.8	1.9 ±0.6	0.156
Surface motive	1.5 ± 0.7	1.9 ± 0.5	1.7 ± 0.8	1.8 ±0.6	0.683
Total score	2.3 ± 0.3	2.5± 0.4	2.5± 0.3	2.3± 0.4	0.5

R-SPQ-2F, Revised Study Process Questionnaire of Two Factors. PGY, postgraduate year

## Discussion

Our study shows the results of anesthesiology residents’ learning approaches and perception of the educational environment in our institution. Our results indicate that there is no correlation between the perception of the learning environment and the approach to learning adopted by anesthesiology residents and that these finding does not change with years of training.

The internal consistency for R-SPQ-2F questionnaire was acceptable with a Cronbach alpha ranging between 0.604 and 0.76 between domains, whereas for the DREEM questionnaire, the internal consistency was questionable (Cronbach alpha of 0.6). We considered that adjustment for inconsistent responses by the exclusion of some questions was necessary for better assessment of these multi-item scales.^24^ The relatively low Cronbach alpha values may be due to the limited number of questions, poor interrelatedness between items or heterogeneous constructs.^25^ We consider that both learning approaches and educational environment present themselves as a continuous heterogeneous construct in residency training, rather than a dichotomous one. In our opinion, the correlation of learning approaches and environment are complex, requiring equally complex non-homogeneous predictors for objective and subjective criteria of evaluation. In other words, the application of undergraduate instruments to evaluate these two important dimensions of medical education, in the context of residency training is an inadequate practice.

Intuition leads researchers to think that the more advanced a student is in years of education, the greater their tendency to adopt deep approaches to learning. Mirghani et al. showed that medical students preferred deep approaches to learning as opposed to a superficial approach preferred by first and second-year students.^26^ Delva and colleagues evidenced that the approach to learning was related to workplace environment when medical students and residents were evaluated with the Workplace Learning Questionnaire and that this correlation varied with years of training. However, there is no mention of an approach to learning in relation to years of education.^27^ Our study did not show a correlation between learning environment perception/approach and years of training for anesthesiology residents. These results may be related to the fact that by the time residents enter their postgraduate education cycle, they have already defined their preferred approach to learning, and that the learning environment does not change significantly during training. On the other hand, we acknowledge that the adoption of a determinate approach to learning by a resident is a dynamic process. Depending on the particular situation, a resident adopts one approach over the other.^28^

We found a moderate correlation between total deep approach scores of the R-SPQ-2F questionnaire and the total subjective perception of teacher’s scores of the DREEM questionnaire. Campbell et al. found that students with deep approaches to learning demonstrate a more sophisticated understanding of the teaching they receive compared to a student with superficial approaches.^29^ The teacher as a role model is a significant contributor to the approach to learning adopted by students. Clinical teacher awareness of their role has significant influence on the teaching-learning experience.^30^ Postgraduate clinical teaching demands for trained trainers.^31^ Some authors advocate for a structured journey that a clinician should follow to become a medical teacher.^32^ Our findings support the notion that the way teaching is conveyed to anesthesiology residents might be related to their learning approach and potentially to learning outcomes.

The 3P model has outlined the complexity of the learning process, that conceives the learning process as a chronological sequence of consecutive phases.^33^^,^^34^ These phases are called presage, process, and product. The presage phase details the personality distinctions between all of the people involved in the learning process.^35^ The process phase describes the specific learning approach or process that both students and teachers utilize in order to either learn or teach information. In the product phase, all of the learning is completed, regardless of the approach, and the evaluation has been graded. A student’s conception of learning, their conception of their teacher, and a student’s individual contextual experience combine to determine their perception of their learning environment. Although the three phases are well defined in undergraduate education, where most of the learning process takes place in a classroom, in residency training the definition of the phases is blurred. In our study, administration of the R-SPQ-2F questionnaire did not discriminate learning approaches per year of training. We might argue that the three phases are experienced in a different way as the resident progresses through training, and an instrument specifically designed to evaluate these dimensions might be necessary to evaluate learning approaches at different levels of training.

Our study has limitations. We evaluated a population of anesthesiology residents of a single program in the United States, limiting the generalizability of our results. Residency programs outside the United States may be structured differently in terms of team-based care. In American residency programs, the post-graduate student receives constant input from diverse sources, including nursing and administrative staff as well as from medical personnel, which has a direct impact on the perception of the educational environment. On the other hand, the differences between general educational systems across countries may account for differences in learning approaches adopted by students. All in all, extrapolation of our results to post-graduate medical programs must be done with caution. In addition, we consider that including residents of different years of training and from different backgrounds provide a rich sample of individuals at different stages of their educational progress. Overall, our results should be taken within the context for a residency program with a size similar to the one at our institution. A Cronbach value of 0.60 was deemed satisfactory. However, values greater 0.70 were considered good, and results should be interpreted with this understanding. Future research is warranted to develop and validate an instrument able to assess learning approaches and perception of the educational environment in postgraduate anesthesiology education.

## Conclusions

The learning approaches adopted by anesthesiology residents and the perception of the educational environment are not correlated with years of training. Additionally, the DREEM and R-SPQ-2F questionnaires are not adequate for evaluation of the correlation between learning environment perception and approaches to learning in anesthesiology residents. Our study has implications for post-graduate education in anesthesiology. The adoption of DREEM and R-SPQ-2F questionnaires that have been validated in general educational contexts, should not be recommended in the post-graduate anesthesiology setting. In addition, future research should focus on the design, development, and validation of instruments to assess the relationship between the learning approach and the perception of educational climate in post-graduate medical training, as well as the effect of progress on these dimensions during the years of residency.

### Conflict of Interest

The authors declare that they have no conflict of interest.
